# Comparative Morphologic and Morphometric Study on the Developmental Aspects of In Vitro and In Vivo Reared Echinococcus granulosus Sensu Stricto Using Differential Interference Contrast (DIC)/Nomarski and Phase Contrast Microscopy

**Published:** 2019

**Authors:** Seyedeh Faezeh SADJJADI, Mina MOTAMEDI, Tahereh MOHAMMADZADEH, Seyed Mahmoud SADJJADI

**Affiliations:** 1. Department of Biology, Faculty of Sciences, Shahid Bahonar University of Kerman, Kerman, Iran; 2. Health Research Center, Life Style Institute, Baqiyatallah University of Medical Sciences, Tehran, Iran; 3. Department of Parasitology, Faculty of Medicine, Baqiyatallah University of Medical Sciences, Tehran, Iran; 4. Department of Parasitology and Mycology, School of Medicine, Shiraz University of Medical Sciences, Shiraz, Iran; 5. Basic Sciences in Infectious Diseases Research Center, Shiraz University of Medical Sciences, Shiraz, Iran

**Keywords:** *Echinococcus granulosus* sensu stricto, G1 strain, DIC/Nomarski microscopy, Phase contrast, Morphology, Morphometry

## Abstract

**Background:**

*Echinococcus granulosus* is a zoonotic parasite with worldwide distribution. The present study focused on comparative morphologic and morphometric observations on the developmental aspects of whole body, more special the reproductive structures of in vitro reared adult worms (RAW) and in vivo reared adult worms in definitive host (AWIDH) using differential interference contrast (DIC)/Nomarski, phase contrast and routine optical microscopy.

**Methods:**

A total number of 10 in vitro and 10 in vivo reared adult worms of *E. granulosus* sensu stricto, G1 strain were selected. The worms were processed by Formaldehyde-Alcohol-Azocarmine-Lactophenol (FAAL). The details of morphological factors and reproductive structures of each worm including 25 biometrical parameters were studied by routine optical, phase contrast and Nomarski microscopy. The details of the samples were photographed, measured and analyzed. The fine structures of the parasite including the details of cirrus sac and developmental stages in different strobila were more obvious observing by Nomarski microscopy.

**Results:**

The morphometric characters in the RAW and AWIDH showed that length of immature proglottid, length of mature proglottid, length of suckers are larger in RAW than AWIDH worms with statistical difference. Characters in *E. granulosus* of RAW and AWIDH showed that total number of segments, number of mature segments and the total number of testes were greater in RAW than AWIDH worms; while only the number of mature segments was statistically different is two groups.

**Conclusion:**

Application of DIC/Nomarski and phase contrast microscopy together with morphometric criteria are useful means for comparing the developmental aspects of in vitro and in vivo reared adults of *E. granulosus*.

## Introduction

E*chinococcus granulosus* is a small zoonotic tapeworm with worldwide distribution (1, 2). Its length is approximately 2 to 7 mm with typically three segments and rarely more than five proglottids. The adult cestodes live in the small intestine of canids where they produce infective eggs which may infect ungulates (sheep, goats, cattle, pigs, horses, etc.) and also humans (3–5). Eating infected offal of the intermediate hosts by the definitive host will complete its life cycle.

So, for prevention of the disease in infected areas, boiling offal containing hydatid cysts for 30 minutes will be very helpful (6). *E. granulosus* sensu lato (s.l.) consists of independent species including *E. granulosus* sensu stricto (s.s.), *E. equinus*, *E. ortleppi*, *E. canadensis and E. felidis* (7). *E. granulosus* (s.s.), *E. ortleppi*, and *E. canadensis* have been reported in Iran, so far (2, 8, 9).

Although major advances have been made in research on *Echinococcus* and echinococcosis, many questions remain, particularly in the areas of developmental biology and host-parasite relationships (3). In this regard, detailed study of adult worms will help a better understanding of the developmental biology of this parasite which could be applicable for treatment and vaccine developments. This is usually done by routine optical microscopy. However, use of routine optical microscopy alone could not be enough for a detailed study of the morphological structure of this parasite. Differential Interference Contrast (DIC) microscopy, also known as Nomarski Interference Contrast (NIC) or Nomarski microscopy which is applicable in biology more special in developmental biology has been used for the present study (10). Imaging modalities, such as differential interference contrast (DIC) microscopy (10, 11) and phase-contrast (PC) microscopy (11), which can capture images of transparent objects are of a significant importance (12).

Many aspects of adult worms of *E. granulosus* have been studied (5, 13–17). However, the morphologic details of in vitro reared adult worms (RAW) and in vivo reared adult worms from the definitive host have not been compared by Nomarski and phase contrast microscopy, so far. Therefore, the present study was designed to compare the morphologic and morphometric criteria of whole body especially developmental aspects of in vitro and in vivo reared *E. granulosus* s.s. adult worms using differential interference contrast (DIC)/ Nomarski and phase contrast and routine optical microscopy.

## Materials and Methods

### In vitro adult worms

In vitro reared adult worms were obtained under aseptic conditions by culturing a number of protoscoleces in culture media. In this regard, sheep hydatid cysts collected in abattoir were dissected for the collection of protoscoleces (PSCs) followed by washing and cultivation in a diphasic S.10E.H medium using a CO2 incubator. Briefly, the surface of the hydatid cysts was washed by 70% ethanol and opened in order to remove Hydatid cyst fluid (HCF) and protoscoleces (PSC) under aseptic conditions. HCF containing PSCs was collected from cysts and transferred into sterile tubes while their PSC were sedimented after 30 min. PSCs were washed 3 times with sterile PBS. The protoscoleces were separated and cultivated under laminar flow hood. The medium was S.10E.H, consisted of 2 parts: liquid phase and solid phase using the same procedures described before with some modifications (13, 15, 18).

The media was removed and substituted by new media each week for two months until the adult worms were produced. The adult worms were removed and transferred into FAAL which for fixing, clearing and staining (19). A number of protoscoleces were also used for molecular works.

### Preparation of samples for routine, phase contrast and DIC/Nomarski microscopy

A total of 10 samples of in vitro reared adult worms were used for microscopic studies. A total of 10 samples of in vivo adult worms from an experimentally infected dog were also used for the present study. Partial cox1 sequence was used to finding their strain which revealed them as *E. granulosus* sensu stricto, G1 strain (20). Routine optical, phase contrast and DIC/Nomarski microscopy were used for the study on the morphological details of each parasite. The detail of each worm was studied separately by descriptive and numerical methods. All aspects of the worms were photographed by a digital camera and prepared for measurement and further analysis.

### Image analysis and statistical analysis

A total of 25 biometrical parameters were observed and measured (Table 1–3).

**Table 1:** Morphometric characters of Adult *Echinococcus granulosus* worm reared in culture media and isolated from definitive host. Significant differences indicate by star. The measurements are in μm. S.D. refers to standard deviation. Min refers to minimum and max to maximum

***Morphometric characters***	***Reared in culture media***		***Isolated from definitive host***		**t *test***
	***N***	***Mean± S.D. (min–max)***	***N***	***Mean± S.D. (min–max)***	
Body length	10	3138.18±695.64 (1931.86–4584.39)	10	2403.52±908.09 (12.96.89–4307.12)	0.057
Maximum width	10	423.54±63.86 (331.50–529.18)	10	470.22±70.94 (395.07–589.18)	0.139
Length of immature proglottid	10	361.93±90.97 (218.18–546.78)	10	237.14±111.05 (78.02–414.91)	0.013*
Width of immature proglottid	10	216.77±52.68 (141.47–297.39)	10	211.16±63.14 (115.51–308.83)	0.832
Length of mature proglottid	10	886.45±177.68(713.99–1314.96)	9	553.31±287.32 (260.36–1020.48)	0.007*
Width of mature proglottid	10	356.98±61.9 (248.10–447.45)	9	293.42±79.08 (169.35–389.91)	0.066
Length of gravid proglottid	9	1174.37±393.50 (187.37–1519.03)	10	1198.10±444.60 (611.30–2105.68)	0.904
Width of gravid proglottid	9	430.52±63.07 (339.47–529.18)	10	470.22±70.94 (395.07–589.18)	0.217
Width of scolex	10	252.06±28.96 (200.53–298.43)	10	231.60±35.88 (166.34–309.34)	0.178
Length of suckers	10	137.77±23.97 (109.50–193.65)	10	107.43±21.09 (62.13–142.55)	0.008*
Width of suckers	10	111.41±18.35 (92.46–144.96)	10	99.07±11.93 (74.86–117.11)	0.092
Length of neck	10	279.74±53.74 (199.20–347.34)	10	278.36±121.37 (146.23–546.27)	0.974
Minimum width of neck	10	142.34±25.73 (101.23–188.55)	10	134.28±34.34 (91.44–181.81)	0.560

**Table 2:** Morphometric characters of reproductive structures in the Adult *Echinococcus granulosus* worm reared in culture media and Adult worm isolated from definitive host. Significant differences indicate by star. The measurements are in μm. S.D. refers to standard deviation; min refers to minimum and max refers to maximum. N is the total examined specimens

***Morphometric characters***	***Reared in culture media***		***Isolated from definitive host***		**t *test***
	***N***	***Mean± S.D. (min–max)***	***N***	***Mean± S.D. (min–max)***	
Length of cirrus sac	10	256.22±29.84 (197.97–309.86)	10	215.60±20.1 (191.55–248–92)	0.002*
Width of cirrus sac	10	69.37±5.98 (57.33–78.52)	10	66.75±13.82 (49.60–92.69)	0.588
Length of ovary	9	239.07±53.56 (175.28–331.50)	3	287.55±54.98 (224.71–326.83)	0.207
Width of ovary	9	93.52±17.34 (69.54–128.56)	3	51.08±7.078 (44.43–58–52)	0.002*
Length of vitelline gland	10	131.66±48.54 (55.91–227.86)	6	119.20±35.08 (75.53–180.04)	0.594
Width of vitelline gland	10	61.65±21.36 (29.64–91.92)	6	67.40±25.21 (42.52–109.91)	0.633
Length of vagina	10	195.44±32.99 (152.27–246.42)	10	245.20±58.88 (156.32–334.91)	0.032*
Width of vagina	10	36.99±7.80 (25.83–50.18)	10	24.87±5.96 (20.15–38.58)	0.001*
Length of seminal receptacle	10	112.49±41.07 (50.30–179.32)	9	77.43±21.48 (41.22–110.70)	0.035*
Width of seminal receptacle	10	68.13±36.27 (35.62–140.28)	9	59.09±17.47 (36.14–88.93)	0.506
Length of egg	_	_	10	27.67±13.84 (11.85–51.61)	_
Width of egg	_	_	10	24.19±13.30 (10.09–48.97)	_

**Table 3:** Descriptive analysis of meristic characters in the Adult *Echinococcus granulosus* worm reared in culture media and isolated from definitive host. Significant differences indicate by star. S.D. refers to standard deviation; min refers to minimum and max refers to maximum; N is the total examined specimens

***Meristic character***	***Reared in culture media***		***Isolated from definitive host***		**t *test***
	***N***	***Mean± S.D. (min–max)***	***N***	***Mean± S.D. (min–max)***	
Total number of segments	10	4.40±0.84 (3–6)	10	3.90±0.31 (3–4)	0.096
Number of mature segments	10	2.00±0.47 (1–3)	10	1.00±0.00 (1–1)	0.000*
Total number of testes	10	32.00±4.81 (25–39)	4	30.75±0.96 (30–32)	0.623

Image-J software base on micron unit was used for image measurements and analysis. Data which obtained from Image-J software were collected in an EXCEL sheet and analyzed by IBM SPSS Statistics 25. The *t*-test was used for statistical differences (P-value<0.05).

## Results

**Fig. 1:**
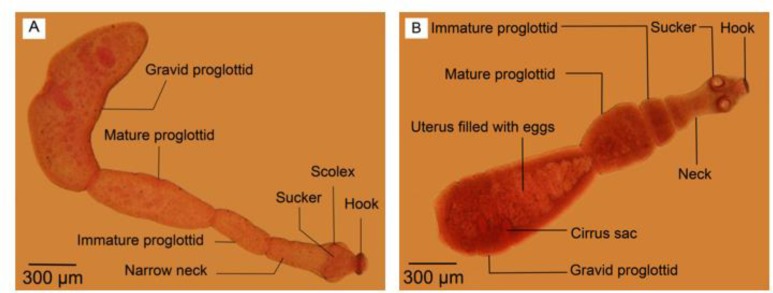
Adult *Echinococcus granulosus*. (A) Reared in culture media. (B) Isolated from definitive host. Scale bars: 300 μm

**Fig. 2:**
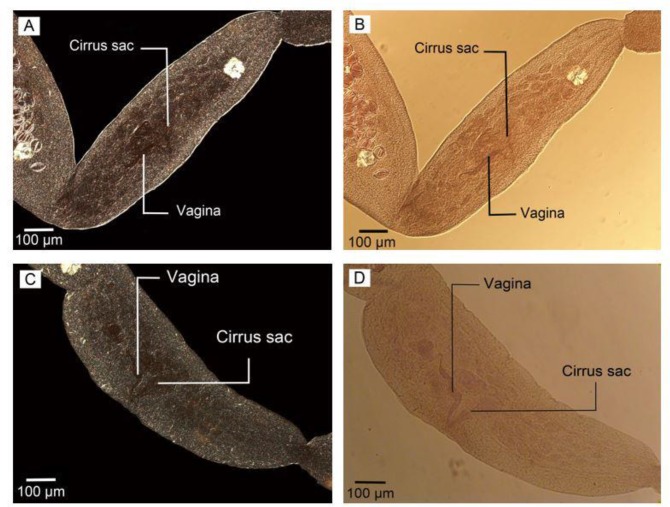
Reproductive structures in mature proglottid in adult worm isolated from definitive host. (A, C) Nomarski microscopy (B) Phase contrast microscopy (D) Optical microscopy. Scale bars: 100 μm

**Fig. 3:**
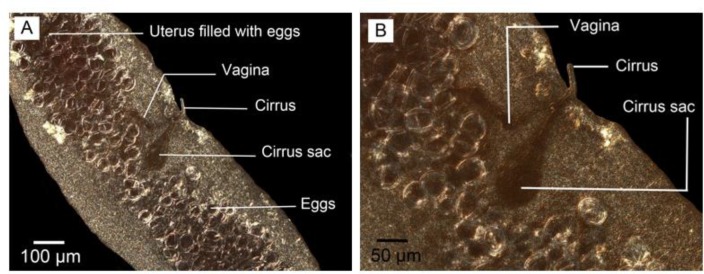
Nomarski microscopy in adult worm isolated from final host. (A) Eggs, cirrus sac and vagina in gravid proglottid. (B) Cirrus sac and Cirrus. Scale bars: (A) 100 μm, (B) 50 μm

The culture of protoscoleces in CMRL media ended with 10 reared adult *E. granulosus* sensu stricto which were fixed, transparented and stained with FAAL. The adult worm samples isolated from a dog experimentally infected with G1 strain of *E. granulosus* were also cleared, fixed and stained with FAAL (Fig. 1). Nomarski microscopy showed suitable details of the samples reproductive structures (Fig. 2–3).

### Descriptive results

Among reared adult worms and adult worms isolated from the definitive host (10 worms from each group), all the worms possess a scolex with two rows of hooks including large and small ones.

Each hook has a hand attached to a blade. A narrow neck attached to the immature proglottid.

**Fig. 4:**
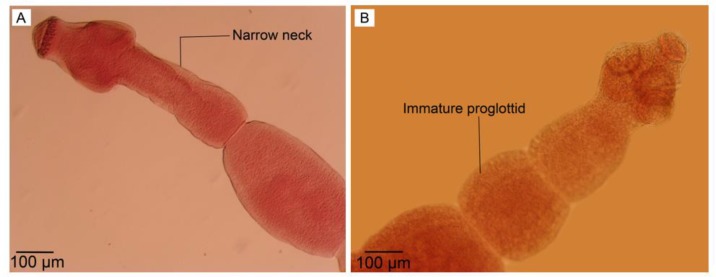
Optical microscopy. (A) Adult worm reared in culture media with narrow neck. (B) Immature proglottid without reproductive structures in adult worm isolated from definitive host. Scale bars: 100 μm

The immature proglottid lacked reproductive structures (Fig. 4).

**Fig. 5:**
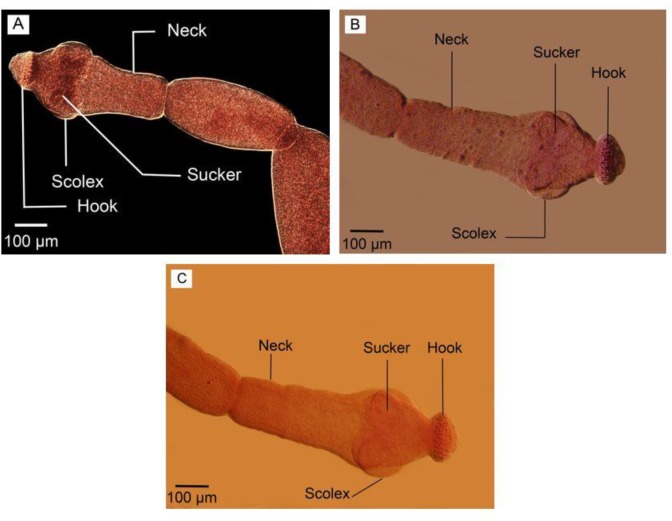
Scolex. (A) Nomaski microscopy. (B) Phase contrast microscopy. (C) Optical microscopy. Scale bars: 100 μm

Both phase contrast and Nomarski microscopy showed the hooks, scolex, eggs, and details of reproductive structures with specific contrast. However, the observed contrast in Nomarski microscopy was sharper than phase contrast microscopy (Fig. 5).

**Fig. 6:**
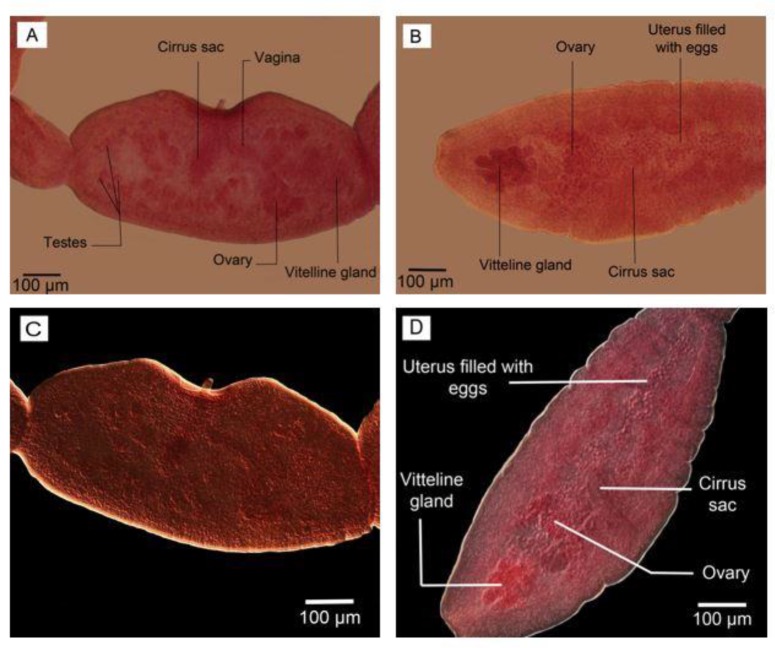
The details of reproductive system in adult worms. (A, B) Phase contrast microscopy; (A) reared in culture media, (B) isolated from definitive host. (C, D) Nomarski microscopy; (C) reared in culture media, (D) isolated from definitive host. Scale bars: 100 μm

The details of the reproductive system with specific contrast are shown in phase contrast microscopy photos (Fig 6. A, B) as well as Nomarski microscopy (Fig. 6. C, D).

**Fig. 7:**
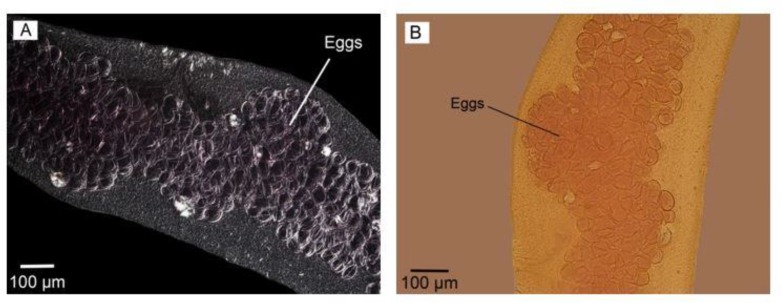
Uterus filled with eggs in gravid segment in adult worm isolated from definitive host. (A) Nomarski microscopy. (B) Phase contrast microscopy. Scale bars: 100 μm

**Fig. 8:**
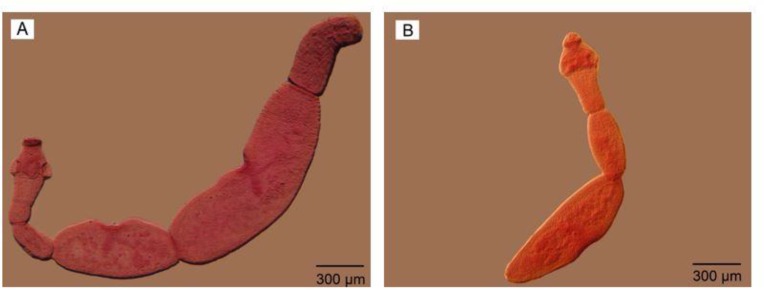
Adult worm reared in culture media (A) 6 segments. (B) 3 segments. Scale bars: 300 μm

The contrast of eggs and cirrus sac were sharper in Nomarski microscopy observation (Fig. 3). The gravid proglottids in reared adult worms were somewhat like the mature segment containing all reproductive structures with uterus a little bigger without eggs. No egg/s was observed in the uterus of adult worms reared in culture media. However, their cirrus sac, vagina, ovary, vitelline gland, and a number of testes were seen clearly; more obvious using phase contrast microscopy (Fig. 6, A). On the other hand, the gravid segment in the adult worms isolated from the final host had a uterus full of eggs which were observed as ovoid-shaped with more obvious structures DIC microscopy observation (Fig. 7). Comparing two types of worms, the maximum number of segments equal to 6 was observed in adult worm reared in culture media (Fig. 8).

### Numerical results

The results of the morphometric study in the adult worms of *E. granulosus* reared in culture media and adult worms of *E. granulosus* isolated from the definitive host are present in three parts including general characteristics, reproductive system and measurable characters which have been shown in Tables 1–3.

The details of morphometric measurements of the reproductive structures in the adult *E. granulosus* worms reared in culture media and adult *E. granulosus* worms isolated from the definitive host are shown in Table 2.

The results of measurable characters like the total number of segments, the number of mature segments and the total number of testes are shown in Table 3. According to this table, the average number of mature segments in RAW was 2.00±0.47 with a range of 1–3 while of AWIDH was 1.00±0.00. Using t-test the difference was significant. The vaginal size was 99.36 in the RAW group and 87.24 in the AWIDH group with a statistically significant difference. Analysis of 12 characters related to the reproductive system between two groups, showed that 5 characters including the length of cirrus sac, the width of the ovary, length of vagina, the width of the vagina, length of the seminal receptacle are a statistically significant difference between two groups (*P*-value ≤0.05).

There were no significant differences between 5 characters related to reproductive structures (Table 2).

## Discussion

Many aspects in the life cycle of *E. granulosus* has been studied in details in recent years and major advances have been achieved on *Echinococcus* and echinococcosis. However, many questions remain, particularly in the areas of developmental biology and host-parasite relationships (3).

In vitro cultivation of *E. granulosus* has been undertaken for the last few decades in order to study the developmental biology, differentiation, host-parasite relationships and using their antigens for vaccination aims (18, 21–23). Moreover, *E. granulosus* parasite can be considered as a model for studying all the developmental stages of the reproductive system in a single adult worm (24). It has been introduced as a new model for parasitologic studies, gene expression studies, stem cell investigation and evolution and developmental biology (25, 26). In vitro cultivation of *Echinococcus* species revealed this method as a suitable differentiation model. In this regard, different morphological stages have been classified and the genes responsible for the main molecular events that lead to structural developments of *E. granulosus* have been investigated (14, 21, 27). However, they did not care about the details of morphology and morphometric criteria in their reports which are one of the main aims of the present study. Our results showed both morphologic and morphometric differences in the in vitro and in vivo reared adult worms. In the previous studies, routine light microscopy has been used for observing the differences. However, simultaneous application of Nomarski (DIC) and phase contrast microscopy have not been used, so far. Application of such instruments in the present study has revealed more obvious and measurable results on this important zoonotic helminth. The mean total number of segments in our study was 4.40±0.84 with a range of 3–6 proglottids in the reared worms which is different by the report of Smyth, 1967 who reported that although in diphasic basic medium (with solid serum) cultured worms underwent segmentation and produced one, two and finally three proglottids (13, 14, 21).

Final maturation resulting in the production of eggs was not achieved as has been reported by others (14, 18, 21, 28). The maximum development of the female genitalia in a proglottid has been observed during in vitro studies and the uterus and vagina have not been tubular but remained as solid cords of cells (21), which are similar to our results.

The failure of the 3-segmented worms to develop completely to maturity in vitro is likely to be a nutritive one due to lack of some growth materials or factors in either the solid or liquid phase (21). However, in vitro culture of *E. granulosus* s.s from protoscolex to the adult stage showed, up to six proglottids in a worm (18).

Studies of in vitro reared *E. multilocularis* also revealed that no shelled eggs were found. Comparative morphological studies on the maturation of the genitalia have been revealed some differences between in vivo and in vitro material. The absence of fertilized eggs in vitro reared worms seems to be due to a variety of factors, rather than just a failure of self-insemination in cultural worms, reflecting possible deficiencies in the culture system (28).

Other studies on in vitro generated *E. granulosus* adult worms have shown similarities with naturally grown worms, with a difference by missing egg production in vitro grown helminths (28). In our study, we used routine optical microscopy, phase contrast microscopy, and Nomarski microscopy to study the samples which stained with FAAL, while in a previous study on *E. granulosus* developmental stages just carmine staining has been used (29).

Comparison of body lengths in RAW and AWIDH groups showed no significant difference. These results are matching with other researches working on mature worms (30, 31). Length of immature proglottid, length of mature proglottid and length of suckers based on Table 1 have shown a significant difference. Several in vitro studies have tried to culture protoscoleces to adult worms with no information on morphometric criteria (18, 27). In a study, routine light microscopy was used for morphometric measurements of strobila of adult worms from dogs experimentally infected with protoscoleces (29). However, using DIC/ Nomarski and phase contrast microscopy has not been applied to the mentioned studies. The greater size of the seminal receptacle was observed in the RAW group which is probably due to the absence of eggs in this group and the not expanded uterus in the gravid proglottid.

Different works on the morphology and morphometry have been carried on adult worm collected from dogs (29–32). The maximum number of segments to be 4. In the same report, the total number of testes was measured to be 37.0 ± 3.94 in the sheep origin worms (32). However, another study on the isolated worms from dogs showed that the total number of segments were 2–3 (31), which is different from our study.

A study on goat-dog specimens showed that the number of segments in the gravid worms was either 3 or 4 (33). Different developmental stages of *E. granulosus* in biphasic culture media has been reported which is lacking the details of developmental criteria of the reared worms (27). A study concluded that adult’s morphology could be genetically determined by the *E. granulosus* s.l. genotype instead of being influenced by the intermediate host of origin (16). This phenomenon needs to be more investigated by molecular studies. Observations on the development of adult *E. granulosus* demonstrated that in addition to ‘normal’ worms, cultures often contained worms that had matured but not segmented (monozoic) and worms with more than one scolex and other malformations (24). This phenomenon was not observed in our study. Previous studies represent anatomical differences in RAW and AWIDH groups (14) which was similar to our study.

## Conclusion

Application of DIC/Nomarski and phase contrast microscopy for details of morphology together with morphometry are useful means for comparing the whole worms, more special in a demonstration of developmental aspects of the reproductive system of RAW and AWIDH worms. To our knowledge, this is the first morphometric comparison between details of RAW and AWIDH worms using DIC/Nomarski and phase contrast microscopy.
